# Joint aspiration for diagnosis of chronic periprosthetic joint infection: when, how, and what tests?

**DOI:** 10.1186/s42836-023-00199-y

**Published:** 2023-09-02

**Authors:** Nicole Durig Quinlan, Jason M. Jennings

**Affiliations:** 1 Colorado Joint Replacement, 2535 S. Downing Street, Ste 100, Denver, CO 80210 USA; 2 Department of Mechanical and Materials Engineering, University of Denver, 2155 E. Wesley Ave, Denver, CO 80210 USA

**Keywords:** Infection, Arthroplasty, Diagnosis, Hip, Knee, Aspiration

## Abstract

Diagnosing chronic periprosthetic joint infection (PJI) requires clinical suspicion in combination with both serological and synovial fluid tests, the results of which are generally applied to validated scoring systems or consensus definitions for PJI. As no single “gold standard” test exists, the diagnosis becomes challenging, especially in the setting of negative cultures or equivocal test results. This review aims to address the workup of chronic PJI and considerations for clinical evaluation to guide treatment. Following aspiration of the joint in question, a multitude of tests has been developed in an attempt to assist with diagnosis, including cell synovial white blood cell count, gram stain, cultures, leukocyte esterase, alpha-defensin, synovial C-reactive protein, multiplex polymerase chain reaction, next-generation sequencing, and interleukins. Each test has advantages and disadvantages and should be used in conjunction with the overall clinical picture to guide further clinical evaluation and treatment in this complex patient population.

## Background

The diagnosis of periprosthetic joint infection (PJI) is rarely as clear-cut as a draining sinus tract and no single, perfect diagnostic test exists. A solitary, “gold standard” test has yet to be established. Thus, multiple consensus groups have developed clinical definitions for PJI with associated scoring systems to assist in diagnosis and guide clinical treatment [1–3]. A variety of serological and synovial fluid tests exist to aid in the diagnosis of periprosthetic joint infection, with some being more beneficial in a chronic infection setting compared to others. The aim of this article is to review the considerations for the workup of chronic PJI and to discuss the advantages and disadvantages of the current tests available to assist in PJI diagnosis in conjunction with clinical findings.

## Overview

Serological testing (i.e., C-reactive protein CRP and erythrocyte sedimentation rate [ESR]) is a ubiquitous test that has been shown to be cost-effective and highly sensitive as a preliminary screening tool in patients with a painful total joint arthroplasty [4–6]. The 2018 ICM minor diagnostic criteria thresholds for chronic PJI are 30 mm/h for ESR and 10 mg/L for CRP [1]. We do not send for serum white blood cell (WBC) and differential analysis since this does little to improve the diagnostic accuracy of PJI [7]. When either the ESR or CRP is elevated, aspiration of the joint is recommended. Additionally, if there is a high index of suspicion for infection in the setting of normal serum laboratory values, performing an arthrocentesis and synovial fluid analysis should be done to rule out PJI [8]. Currently, there are no guidelines for the ideal “timing” of an aspiration to rule out chronic PJI. It is the authors’ opinion that any elevation in serological testing and/or a high clinical index of suspicion warrants an aspiration. Furthermore, many of the tests discussed below may assist when “standard” tests are equivocal.

Aspiration of the knee is performed employing the standard sterile technique via a superolateral approach. In patients with excess soft tissue around the knee, we recommend the use of a spinal needle to ensure that adequate depth is achieved to obtain the synovial fluid sample. In rare circumstances, ultrasound or fluoroscopic guidance may be necessary for the aspiration to achieve a sufficiently-sized sample of fluid. It is recommended that hip aspirations are performed under image guidance (i.e., ultrasound or fluoroscopy).

The manufacturing guidelines should be referenced for each planned test prior to aspiration to ensure ample fluid is acquired. For commercially available tests, the amount of synovial fluid required ranges from 20 µL to 2 mL depending on the desired test. The minimum amount for alpha-defensin and cell count at most laboratories is 0.5 mL. Next-generation sequencing often requires 2 mL of fluid. Rockov et al. found that higher aspiration volumes are more likely to correlate with intraoperative cultures and that an even higher volume was required for slow-growing microorganisms. This study reports that at least 1 mL is required to send for culture, though 5 mL allows for gram stain, fungal stain and acid-fast bacillus stain to all be run, and above 10 mL the sensitivity of each of these cultures is maximized. The optimal volume cutoff for concordant intraoperative cultures was 3.5 mL for typical organisms and 12.5 mL for slow-growing organisms [9]. If aspiration is performed such that blood is included in the sample, a collection tube containing ethylenediaminetetraacetic acid (EDTA), commonly used for complete blood counts (CBC), may be useful to limit clot formation or aberrant test results. Finally, special considerations should be observed in the setting of metallosis as metal debris may alter the results of certain laboratory values. If minimal or no synovial fluid is obtained, we recommend against the addition of saline since this may reduce the sensitivity of traditional laboratory values and contemporary synovial fluid biomarkers [10].

When synovial fluid is obtained, it should be sent for synovial fluid WBC count with differential and conventional culture, including both aerobic and anaerobic analyses. The synovial WBC count and differential analysis have been shown to perform well in the diagnosis of PJI [11]. In the setting of chronic infection, the threshold for minor diagnostic criteria, as defined by the 2018 International Consensus Meeting (ICM) to indicate a high likelihood of infection, is ≥ 3,000 cells/µL for synovial WBC count and ≥ 80% for the percentage of polymorphonuclear neutrophil (PMN) cells in the synovial cell differential [1]. The 2018 ICM thresholds for the minor diagnostic criteria have been included in Table 1 [1]. While false elevation in automated synovial WBC count has been detected in the setting of hip corrosion, there appears to be a substantial risk with all patients with THA or TKA. It has been suggested that clinicians should exercise caution when interpreting elevated automated synovial WBC count and consideration should be given to requesting a manual synovial WBC count to verify the accuracy of the automated cell count [12]. We typically do not rely on gram stain since the sensitivity as a screening tool has been shown to be poor [13–15]. All cultures should be kept in the laboratory for at least 14 days to allow for sufficient time for indolent infection (i.e., *Cutibacterium acnes*) growth. Fungal and acid-fast bacillus (AFB) cultures may also be sent as clinically indicated, particularly in immunocompromised patients or those with persistent infections. Studies have found that diabetes, prolonged use of antibiotics, prior PJI, and immunosuppression are among the risk factors for fungal PJI specifically [16]. When multiple risk factors exist and in patients with poor host immunity, evaluation of synovial fluid for fungal infection is recommended, including the use of fungal-specific culture mediums and a longer incubation time of at least 14 days [16]. The detection of fungal PJI with systemic serum markers and synovial WBC count remains a diagnostic dilemma [17].

**Table 1:** The threshold for the minor diagnostic criteria according to 2018 ICM [1]

**Criterion**	**Chronic PJI (> 90 days) threshold**
Erythrocyte sedimentation rate (mm/h)	30
C-reactive protein (mg/L)	10
Synovial white blood cell count (cells/µL)	3,000
Synovial polymorphonuclear neutrophil (%)	80

In addition to standard synovial fluid tests, several currently available synovial biomarkers are also available, which may be beneficial in the diagnosis of PJI, and are described in more detail below and summarized in Table 2.

**Table 2:** Summary of synovial fluid biomarkers

**Biomarker**	**Efficacy**	**Amount of Fluid Required (mL)**^**a**^	**Wait Time**^**a**^	**Advantages**	**Disadvantages**	**Ref.**
Leukocyte esterase	Sensitivity: 49%–95% Specificity: 82%–100% PPV: 72%–100%	~ 20 µL	10 min	Readily available, easy to use, inexpensive Can be used in the setting of recent antibiotics	Can be influenced by presence of blood or metallic debris	[18–24]
Alpha-defensin	Sensitivity: 65%–95% Specificity: 87%–99.6% PPV: 75%–98% NPV: 84%–98%	0.5 mL	Lateral flow test: 10 min ELISA: 2–5 days	Not influenced by recent antibiotic use	Can be influenced by metallic debris and crystalline arthropathies	[18, 25–30]
Synovial CRP	Sensitivity: 70%–96% Specificity: 85%–100% PPV: 68% NPV: 95%	0.5 mL	Approx 1–3 h	Can be used in the setting of recent antibiotic use and inflammatory arthropathies	No information regarding organism	[2, 31–34]
Multiplex PCR	Sensitivity: 85% Specificity: 98% PPV: 97.6% NPV: 87.5%	180 µL	Approx. 5 h	Provides causative organism and potential antibiotic resistance Can be used in the setting of recent antibiotic use	Not readily available Causitive organism must be present on the cassette in order to be detected	[25, 35, 36]
Next generation sequencing	Sensitivity: 89%	2 mL	In house: 9–48 h Send out: 2–7 days	Provides causative organism and potential antibiotic resistance Can be used in setting of recent antibiotic use and inflammatory arthropathies Most useful in culture-negative cases	Not readily available, costly Requires specialized equipment or lab send-out High rate of contamination and false positives	[37–42]
Interleukins	Sensitivity: 100% Specificity: 100%	110 µL	Lateral flow: < 20 min Immuno-assay: approx. 1–5 h	Can be used in the setting of recent antibiotic use	Accuracy is dependent on immunoassay technique	[18, 43]
Calprotectin	Sensitivity: 71%–94% Specificity: 81%–88% PPV: 0.71–0.77 NPV: 0.76–0.98	20 µL	15 min	Fast turn-around Requires smartphone application only for analysis	No definitive thresholds for PJI at this time Not yet widely available	[44–47]
Neutrophil gelatinase-associated lipocalin	Sensitivity: 86%–100% Specificity: 77%–100%	0.5 mL	15 min to 4.5 h	Can be used in the setting of recent antibiotic use and inflammatory arthropathies	No definitive thresholds for PJI at this time May be correlated with number of neutrophils present in sample	[48–52]
Lactoferrin	Sensitivity: 97%–100% Specificity: 90%–100%	0.5 mL	In house: 4–24 h Send out: 4–8 days	Can be used in the setting of inflammatory arthropathies	No definitive thresholds for PJI at this time May be correlated with number of neutrophils present in sample Minimal clinical studies available	[46, 51, 53]

^a^Manufacturing guidelines on company websites or the physical test kit pamphlets were used to determine these values, which can be largely variable depending on the manufacturer. Variations in wait time should be considered as these are largely dependent on specific laboratory equipment and capabilities

### Leukocyte esterase

Leukocyte esterase (LE) is an enzyme produced by activated neutrophils in response to inflammation or infection. The test results are available instantaneously by reading a colorimetric reagent test strip that has been exposed to synovial fluid. Studies have demonstrated a sensitivity of 49%–95%, a specificity of 82%–100%, and a positive predictive value ranging from 72%–100% [18, 19]. The test is readily available, technically easy to perform and inexpensive, making it an ideal point-of-care test for PJI. A “clean” synovial fluid sample (i.e., without blood or metallic debris) is unlikely to yield false positive results [20]. It appears that the LE strip test can be utilized as a reliable diagnostic tool for the diagnosis of PJI even when prior antibiotics have been administered [21, 22]. Additionally, it may serve as a screening tool to rule out PJI in patients with failure of THA secondary to metal particle release [23]. However, the major disadvantage of this test is that results may be influenced by the presence of blood or metal debris within the synovial fluid sample. When blood is present, the centrifuge may be utilized to help maintain the reliability and accuracy of the test [54]. Its overall performance has made it a valuable part of the 2018 ICM criteria [24].

### Alpha-defensin

Alpha-defensin (AD) is an antimicrobial peptide produced naturally by neutrophils in the presence of bacterial pathogens. Ranges for sensitivity, specificity, positive predictive value (PPV) and negative predictive value (NPV) have been cited at 65%–95%, 87%–99.6%, 75%–98% and 84%–98%, respectively [18]. While AD lateral flow immunoassay techniques are faster, some studies suggested that sensitivity, specificity and PPV are improved when the test is performed in the laboratory by enzyme-linked immunosorbent assay (ELISA) [18, 25], while others demonstrated no difference in accuracy when comparing the two techniques [26]. One potential disadvantage of AD is that the diagnosis accuracy is decreased in the presence of metallosis with adverse local tissue reaction (ALTR) [27, 28] and in crystalline arthropathies [29].

The diagnostic accuracy of AD for PJI diagnosis is comparable but not superior to that of synovial WBC count and percent PMN combined [55]. Additionally, AD has not been shown to change the clinical management of patients being worked up for PJI or in the setting of reimplantation of chronic PJI when compared with traditional laboratory values [56–58]. These studies questioned the routine use of this synovial biomarker. Lastly, premature antibiotic administration in the setting of PJI may compromise the sensitivity of traditional diagnostic laboratory results. When antibiotics have been administered, AD has been shown to have a higher sensitivity and provide better screening for chronic PJI than ESR, CRP, synovial fluid neutrophil percentage and synovial fluid culture [30]. However, no statistically significant improvement was noted over synovial WBC count. As such, its greatest utility may be for equivocal cases as an adjunct for the diagnosis of PJI.

### Synovial CRP

Parvizi et al. [31] originally demonstrated that the synovial biomarker was more accurate in the diagnosis of PJI than its serum counterpart. Some studies have indicated that synovial CRP may have higher sensitivity and specificity for PJI compared to serum CRP [32]. However, others have shown that it has similar sensitivity, specificity and positive predictive value, providing no additional diagnostic advantage compared with serum CRP [33]. Recent studies suggested that the combination of both synovial and serum CRP might improve the diagnostic value in the prediction of PJI, especially in the setting of chronic infection [59, 60]. Lastly, Deirmengian et al. demonstrated increased sensitivity and specificity (97% and 100%, respectively) with the combination of synovial CRP and alpha-defensin, even in complex patient populations with systemic inflammatory disease or recent antibiotic use [34]. While synovial CRP alone may not be sufficient to diagnose PJI, it is included in the validated workup by Parvizi et al. [2] and its inclusion in the workup as an adjuvant should be considered.

### Multiplex polymerase chain reaction

Multiplex polymerase chain reaction (mPCR) is a test that provides the genotypic evaluation of bacteria of a sample specimen. Lausmann et al., by using mPCR, have demonstrated a sensitivity of 85%, specificity of 98%, PPV of 97.6% and NPV of 87.5%, with an overall accuracy of 91.8% [25]. Other studies have also shown improved sensitivity and specificity compared to conventional cultures alone, yet mPCR in combination with traditional cultures provides the greatest diagnostic accuracy [35, 36]. The major advantage of this test is that it provides information regarding the presence of infection as well as the causative organism for the infection and potential antibiotic resistance markers regardless of concurrent antibiotic use [25]. By using this test, the simultaneous detection of an entire panel of potentially causative organisms is possible, even with limited synovial fluid obtainable via aspiration [35]. Additionally, the test results are typically available within approximately 5 h compared to several days with traditional culture results. Some studies have suggested that it may be superior to traditional culture in the detection of low-virulence organisms and have the added advantage of requiring only 180 µl of fluid for evaluation [25, 36]. A limitation of this test is that if the proper primer for the causative organism is not present on the cassette, the test result will be negative and a diagnosis of PJI may be missed [25, 36]. The test is also not readily available at all institutions.

### Next-generation sequencing

Next-generation sequencing (NGS) is a technique by which all of the DNA in a sample specimen is detected and sequenced to determine the presence of specific microorganisms [61]. This technique is still being investigated but offers the advantages of being able to detect multiple causative organisms and determine the presence of possible antibiotic resistance markers [37–39]. Additionally, the data are accurate regardless of antibiotic use by the patient and the presence of systemic inflammatory disease. In particular, NGS may be most useful in patients with culture-negative aspiration results [38, 39]. A recent review demonstrated that the sensitivity of NGS at identifying organisms may be greater than conventional culture, with rates up to 89% in culture-negative PJI [40]. However, Kildow et al. reported improved sensitivity and specificity for PJI detection using traditional culture compared to NGS and mPCR [41]. More recent reductions in the cost of this test, as well as improvements in techniques and the hardware required to run the test have made NGS more accessible for clinical application [40]. Torchia et al. reported that the cost-effectiveness of NGS for PJI diagnosis is dependent upon the pretest probability of PJI and the performance of the technology used for the test, suggesting that it should ideally be reserved for patients with a high pretest probability of infection [42]. Certain NGS methods are also available and the detection of multiple microbe types (bacteria, fungi, parasites, and viruses) by using the methods is possible [40]. While this test offers multiple advantages, a major limitation is the high rate of false positives due to low specificity and high risk of contamination [40]. Additionally, the run time of 2–5 days should also be considered, though this time may be less if facilities have in-house NGS capabilities.

### Interleukins

Interleukins (IL) are inflammatory cytokines that are currently under investigation for use as a diagnostic tool for PJI. IL-6 is the most cited at this time and has the advantage of maintaining accuracy even in patients actively taking antibiotics. There are equivocal results for its use in those with systemic inflammatory disease. Synovial cutoff values are still being studied but values > 9,000 pg/mL have shown a specificity of nearly 100% [43]. Some studies have demonstrated increased positive predictive value when IL-6 is used in combination with serum CRP [43]. The accuracy of the results also depends on the immunoassay technique used, with lateral flow being less accurate than radioimmunoassay techniques [18]. Interleukins as a diagnostic test can be considered for inclusion in the workup of PJI, though further studies are likely needed to evaluate the utility and appropriate settings for the use of these particular biomarkers.

### Calprotectin

Calprotectin is a pro-inflammatory protein released from activated neutrophils and macrophages during inflammation. It has been studied as a potential synovial fluid biomarker for the diagnosis of chronic PJI. Using a lateral flow assay, expeditious analysis can be used intraoperatively for calprotectin, affording the ability to make immediate intraoperative decisions during revision surgery [44]. The lateral flow assay is made available by a company in Norway and photometrically evaluates 20 µL sample after a 15-min waiting period via a smartphone application provided by the manufacturer. The application provides a quantitative value of calprotectin and proposes 3 different risk stratifications for the assessment of the risk of PJI [44]. An additional advantage of this biomarker is its consistent accuracy in the setting of other etiologies for inflammation, including inflammatory arthritis, fracture, dislocation, or recent surgery that may mimic infection [45, 46]. The sensitivity of the test has been reported to be at 71%–94% and the specificity at 81%–88%, with a PPV of 71%–77% and an NPV of 76%–98% [44, 45, 47]. These test kits are not yet widely available in the USA and a specific threshold has not been determined to definitively rule in or out infection. Future studies are warranted to further evaluate this biomarker, but current research suggests it should be considered moving forward, especially in the setting of other etiologies of inflammation.

### Neutrophil gelatinase-associated lipocalin

Neutrophil gelatinase-associated lipocalin (NGAL) is an antibacterial peptide that affects the iron ion metabolism of pathogens and is secreted by neutrophils during an inflammatory response [48]. It is measured using ELISA techniques and only 0.5 mL of synovial fluid is required. A specific threshold that defines infection has not yet been determined and the levels of NGAL may be highly dependent on the number of neutrophils present in the sample [48]. Studies have reported a sensitivity of 86%–100% and a specificity of 77%–100% [48–51]. One advantage of this biomarker is its efficacy in the setting of recent antibiotic, use according to Huang et al. [48]. Further research is necessary to determine appropriate cutoff values to define PJI and clinical settings in which NGAL is most appropriate for use.

### Lactoferrin

Lactoferrin is a glycoprotein involved in the iron metabolism in pathogens, is secreted by neutrophils in the early inflammatory response and plays a complex role in the immune cascade [53]. The biomarker is typically analyzed using ELISA or multiplex PCR techniques, though specific cut-off values have not been determined to definitively rule in or out PJI. Studies have reported a sensitivity of 97%–100% and a specificity of 90%–100% [51, 53]. Wang et al. also found correlations of synovial fluid WBC/PMN percentage with lactoferrin values, which may suggest it is dependent on the number of neutrophils within the sample collected [53]. Studies have found it is a reliable test for the detection of PJI even in patients with inflammatory arthropathies [46], but it has not been widely tested in other clinical scenarios, including in the setting of recent antibiotic use. Clinical studies to further evaluate the efficacy of this biomarker are needed, as well as studies to determine appropriate clinical scenarios for its use. Lastly, there are many other synovial fluid biomarkers, including, tumor necrosis factor-alpha, interferon-gamma, lactate and others, that show promise that is not readily available for use at all institutions and may prove to be advantageous but future studies are needed before these are widely endorsed [62–65].

## Conclusions

The diagnosis of periprosthetic joint infection in the setting of equivocal test results, negative cultures or chronic, indolent infection can raise significant dilemmas, especially without a single, “gold standard” diagnostic test available. The presence of metallosis, inflammatory arthropathies, other concurrent inflammatory processes, recent antibiotic use and other clinically-related factors should be considered when deciding which synovial test is most appropriate. Each test provides valuable information to help guide the surgeon in their evaluation of patients with possible chronic PJI, but the accuracy of such tests is variable and no single test can be relied upon for definitive clinical decisions. These tests should be utilized as adjunct data and, as such, a combination of appropriate tests based on the clinical scenario may be most valuable. However, an exact combination of tests has yet to be determined to most accurately diagnose chronic PJI. Validated scoring systems and consensus definitions of periprosthetic infection [1–3] in conjunction with the overall clinical picture should guide the clinical evaluation and treatment of patients presenting with clinical findings concerning infection.

## Data Availability

Not applicable.

## Abbreviations

PJI: Periprosthetic joint infection
CRP: C-reactive protein
ESR: Erythrocyte sedimentation rate
WBC: White blood cell
EDTA: Ethylenediaminetetraacetic acid
CBC: Complete blood count
ICM: International Consensus Meeting
PMN: Polymorphonuclear neutrophil
AFB: Acid-fast bacillus
LE: Leukocyte esterase
AD: Alpha-defensin
PPV: Positive predictive value
NPV: Negative predictive value
ELISA: Enzyme-linked immunosorbent assay
ALTR: Adverse local tissue reaction
mPCR: Multiplex polymerase chain reaction
NGS: Next-generation sequencing
IL: Interleukins
NGAL: Neutrophil gelatinase-associated lipocalin
